# High-flow Nasal Cannula versus Conventional Ventilation in Laryngeal Surgery: A Systematic Review and Meta-analysis

**DOI:** 10.7759/cureus.38611

**Published:** 2023-05-05

**Authors:** Kai Chun Chan, Timothy Xianyi Yang, Kin Fai Khu, Ching, Vincent So

**Affiliations:** 1 Department of Anaesthesiology and Operating Theatre Services, Queen Elizabeth Hospital, Kowloon, HKG; 2 Department of Anaesthesiology, Princess Margaret Hospital, Kowloon, HKG; 3 Department of Anaesthesiology, Queen Mary Hospital, Hong Kong Island, HKG

**Keywords:** thrive, microlaryngeal surgery, shared airway, laser microsurgery for laryngeal benign and malignant tumors and for airway stenosis, high-frequency jet ventilation, high flow nasal cannula (hfnc)

## Abstract

High-flow nasal cannula (HFNC) is an emerging option for maintaining oxygenation in patients undergoing laryngeal surgery, as an alternative to traditional tracheal ventilation and jet ventilation (JV). However, the data on its safety and efficacy is sparse. This study aims to aggregate the current data and compares the use of HFNC with tracheal intubation and jet ventilation in adult patients undergoing laryngeal surgery. We searched PubMed, MEDLINE (Medical Literature Analysis and Retrieval System Online, or MEDLARS Online), Embase (Excerpta Medica Database), Google Scholar, Cochrane Library, and Web of Science. Both observational studies and prospective comparative studies were included. Risk of bias was appraised with the Cochrane Collaboration Risk of Bias in Non-Randomized Studies - of Interventions (ROBINS-I) or RoB2 tools and the Joanna Briggs Institute (JBI) Critical Appraisal Checklist for case series. Data were extracted and tabulated as a systematic review. Summary statistics were performed. Meta-analyses and trial sequential analyses of the comparative studies were performed. Forty-three studies (14 HFNC, 22 JV, and seven comparative studies) with 8064 patients were included. In the meta-analysis of comparative studies, the duration of surgery was significantly reduced in the THRIVE (Transnasal Humidified Rapid-Insufflation Ventilatory Exchange) group, but the number of desaturations, need for rescue intervention, and peak end-tidal CO_2_ were significantly increased compared to the conventional ventilation group. The evidence was of moderate certainty and there was no evidence of publication bias. In conclusion, HFNC may be as effective as tracheal intubation in oxygenation during laryngeal surgery in selected adult patients and reduces the duration of surgery but conventional ventilation with tracheal intubation may be safer. The safety of JV was comparable to HFNC.

## Introduction and background

Trans-nasal humidified rapid insufflation ventilatory exchange (THRIVE) delivers warm and humidified oxygen through high-flow nasal cannula (HFNC) at a high flow rate of up to 120L/min to patients who are both spontaneously ventilating or apneic. This has been used in both adult and neonatal intensive care units for respiratory distress syndrome [1-3], as an alternative non-invasive ventilatory option for patients with congestive heart failure and pulmonary oedema [2,3], in the peri-operative setting for pre-oxygenation [4], intra-operatively in the setting of difficult intubation [5], in post-extubation [6,7], and in sedation for endoscopies [8-10]. 

There are many modes of conventional ventilatory options during laryngopharyngeal surgery, including mechanical ventilation (MV) with tracheal intubation (TI), superimposed high-frequency jet ventilation (SHFJV) through the supraglottic, infraglottic, and transtracheal routes. Tracheal intubation has traditionally been performed for airway surgery, and intermittent intubation and cessation of oxygenation and ventilation can increase the risk of hypoxemia and carbon dioxide (CO_2_) accumulation with progressive respiratory acidosis. Furthermore, intubation can obstruct the surgical view, hinder surgical access, and prolong surgical duration. There have been many studies on the use of jet ventilation for laryngeal surgery, most of them case series [11-31], which show complications including hypoxia, hypercapnia, need for intubation, and barotrauma. HFNC depends on the patient having a patent upper airway, which is provided by suspension laryngoscopy during laryngopharyngeal surgery. It avoids disturbing the surgical field, facilitates surgical access, and theoretically shortens procedure time.

Since Huang’s review of the use of THRIVE in apneic patients undergoing shared airway surgery [5], there have been more recent studies comparing HFNC to conventional ventilation strategies such as tracheal intubation and SHFJV in the setting of laryngologic surgery [32-35], but there has not yet been a comprehensive comparative systematic review and meta-analysis of each method’s efficacy. This study aims to compare the safety and efficacy of HFNC to conventional ventilation using tracheal intubation or jet ventilation during laryngeal surgery.

Study design and methodology

We conducted a systematic review of the current literature to compare the efficacy of HFNC and conventional ventilation including TI and jet ventilation for laryngeal surgery in adults, and performed a meta-analysis and trial sequential analysis (TSA) on the comparative studies, following the Preferred Reporting Items for Systematic Reviews and Meta-Analyses (PRISMA) guidelines and Cochrane Handbook for Systematic Reviews of Interventions [36]. This study was pre-registered with the International Prospective Register of Systematic Reviews (PROSPERO) (CRD42022311505) and OSF.io (Registration DOI: 10.17605/OSF.IO/DR8M9) on March 3, 2022. 

Inclusion and Exclusion Criteria

Due to the limited number of comparative and prospective studies, we included both retrospective and prospective studies of the use of HFNC and jet ventilation in laryngeal and airway surgery. For the jet ventilation studies, we included supraglottic, infraglottic, and transtracheal approaches. We included randomized controlled trials, non-randomized comparative studies, case series (of three or more patients), and retrospective cohort studies. We included studies of laryngeal surgery, such as microlaryngeal surgery, suspension laryngoscopy, laryngotracheal surgery, vocal cord surgery, and subglottic surgery, including those which utilized laser and robotic surgery. We excluded studies of dental surgery, tracheal reconstruction, bronchoscopy, endoscopic bronchial ultrasound and biopsy, gastroscopy, colonoscopy, endoscopic retrograde cholangiopancreatography, sedation, monitored anaesthesia care for non-airway surgery, HFNC for pre-oxygenation, and induction of anaesthesia only or for intubation. Only traditionally published, indexed journal articles were included. We excluded case reports of single patients, studies that did not report the primary outcomes, and grey literature such as non-traditional articles including expert opinions, audits, editorials, commentaries, conference reports, and abstracts. Articles not written in English were excluded. We excluded studies prior to 2000 for jet ventilation studies to avoid bias of comparing older studies to modern ones. 

Search Strategy

**Figure 1:**
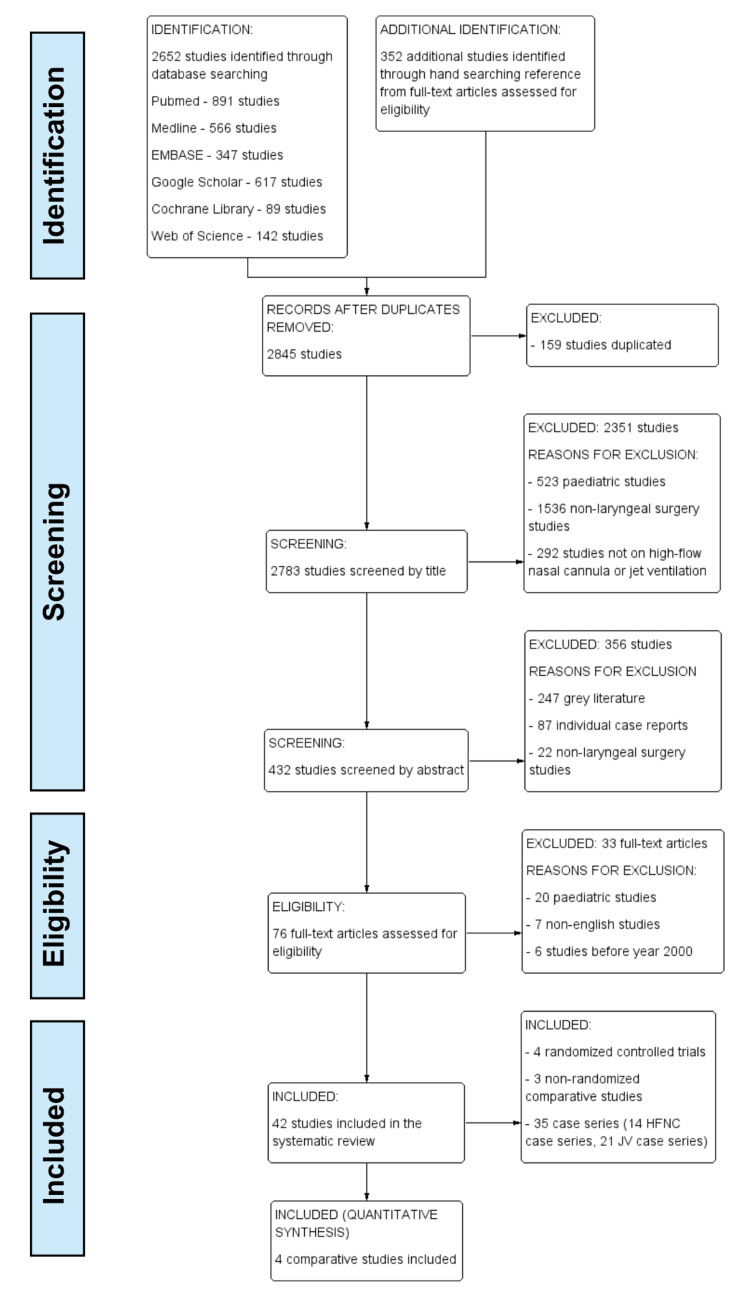
PRISMA flow diagram

Appropriate keywords were identified by a free-hand search. In February 2022, we searched the electronic databases of PubMed, MEDLINE (Medical Literature Analysis and Retrieval System Online, or MEDLARS Online), Embase (Excerpta Medica Database), Google Scholar, Cochrane Library, and Web of Science for relevant results, using a matrix of specific keywords and terms, including “Humans”, “Adult”, “High-Flow Nasal Cannula”, “High Flow Nasal Therapy”, “Nasal Highflow”, “High Flow Nasal Oxygen”, “High Flow Oxygen Therapy”, “Optiflow”, “THRIVE”, “Transnasal Humidified Rapid-insufflation ventilation”, “ Ventilation”, “Laryngeal surgery”, “Airway surgery”, “High Flow Jet Ventilation”, “Jet ventilation”, “High-frequency Jet Ventilation”, “Jet insufflation” and other keywords. Two authors (KC and TY) independently performed title and abstract screening and all initial included papers had their reference lists searched for further results. The PRISMA study flow diagram is presented in Figure 1. 

Risk of Bias Assessment

We used the Joanna Briggs Institute Critical Appraisal Checklist for Case Series to critically appraise the case series [37] (Table 1). A score of 7 or more was high quality, a score of 5-7 was medium quality, and a score of less than 5 was considered low quality. Two authors (C and TY) independently assessed the included studies and evaluated them using the Cochrane Collaboration Risk of Bias version 2 (RoB2) or Risk of Bias in Non-Randomised Studies - of Interventions (ROBINS-I) [38,39] to assess the risk of bias (Tables 2-3). We categorised the risk of bias as high risk, low risk, or unclear.

**Table 1:** Joanna Briggs Institute critical appraisal tool for case series NA = Not applicable; + = yes; - = no; ? = unclear

Study	Clear inclusion criteria	Standard, reliable measurements	Valid methods of identification of the condition in included participants	Consecutive inclusion	Complete inclusion of participants	Clear reporting of demographics	Clear reporting of clinical information	Outcomes and follow up results clearly reported	Clear reporting of presenting site and demographic information	Appropriate statistical analysis	Overall rating of bias Score < 5: low quality Score 5-7: medium quality Score >/= 7: high quality
Benninger et al. (2021) [40]	+	+	NA	-	+	+	+	+	+	+	8
Booth et al. (2017) [41]	+	+	NA	+	-	+	+	+	?	+	6
Booth et al. (2020) [42]	+	+	NA	+	+	+	+	+	-	+	8
Ebeling and Riccio (2019) [43]	-	-	NA	-	+	+	+	+	NA	+	4
Flach et al. (2019) [44]	+	+	NA	+	+	+	+	+	NA	?	7
Yang et al. (2019) [45]	+	+	NA	+	+	+	+	+	+	+	9
Huang et al. (2019) [46]	+	+	NA	+	+	+	+	+	?	?	7
Lyons and Callaghan (2017) [47]	-	+	NA	+	+	+	+	+	NA	+	6
Maupeu et al. (2018) [48]	+	+	NA	+	+	+	+	+	-	+	8
Patel and Nouraei (2015) [49]	+	+	NA	+	+	+	+	+	+	+	9
Rutt et al. (2022) [50]	+	+	NA	+	+	+	+	+	+	?	8
To et al. (2017) [51]	+	+	NA	+	+	+	+	+	+	+	9
Waters et al. (2019) [52]	-	+	NA	+	+	+	+	+	-	+	7
Leiter et al. (2012) [23]	+	+	NA	+	+	+	+	+	+	+	9
Barakate et al. (2010) [15]	+	-	NA	+	-	-	+	+	-	+	5
Bacher et al. (2000) [14]	+	+	NA	-	+	+	+	+	+	+	8
Mora et al. (2017) [24]	+	+	NA	+	+	+	+	+	+	+	9
Patel and Rubin (2009) [26]	+	+	NA	+	+	-	-	+	-	-	4
Rezaie Majd et al. (2006) [28]	+	+	NA	+	-	+	+	+	+	+	8
Halmos et al. (2020) [18]	+	+	NA	+	+	+	+	+	+	+	8
Lanzenberger-Schragl et al. (2000) [22]	+	+	NA	-	-	+	+	+	+	+	7
Bacher et al. (2000) [13]	-	+	NA	-	-	-	+	+	-	+	4
Rubin et al. (2005) [30]	+	+	NA	+	+	+	+	+	-	+	8
Bourgain et al. (2001) [16]	-	+	NA	+	+	+	+	+		+	7
Altun et al. (2018) [12]	+	+	NA	+	-	+	+	+	+	+	8
Davies et al. (2009) [17]	+	+	NA	+	-	+	+	+	+	+	8
Krespi et al. (2019) [21]	+	+	NA	+	+	+	+	+	+	-	8
Jaquet et al. (2006) [20]	+	+	NA	+	+	+	+	+	+	+	9
Putz et al. (2022) [27]	+	+	NA	+	+	+	+	+	+	+	9
Helmstaedter et al. (2015) [19]	+	+	NA	+	+	+	+	+	-	-	7
Russell et al. (2000) [31]	+	+	NA	+	-	+	+	+	+	+	8
Abad et al. (2007) [11]	+	+	NA	+	+	+	+	+	+	+	9
Ross-Anderson et al. (2011) [29]	+	+	NA	+	+	-	+	+	-	+	7
Orloff et al. (2002) [25]	+	+	NA	+	+	-	-	-	-	-	4
Gustafsson et al. (2017) [53]	+	+	NA	+	+	+	+	+	+	+	9

**Table 2:** Risk of bias assessment for randomized controlled trials version 2 (RoB 2) + = yes; - = no; ? = unclear

Study	Random sequence generation	Allocation concealment	Blinding of participants and personnel	Blinding of outcome assessment	Incomplete outcome data	Selective reporting	Other bias
Forsberg et al. (2020) [32]	+	?	-	?	?	-	?
Min et al. (2022) [33]	+	+	+	?	?	?	?
Nekhendzy et al. (2020) [34]	+	?	+	+	-	?	?
Rajan et al. (2018) [54]	+	+	-	?	-	-	?

**Table 3:** Risk of bias assessment in non-randomized studies - of interventions (ROBINS-I) + = yes;  - = no; ? = unclear

Study	No confounding bias	No selection Bias	No classification of intervention bias	No bias due to deviations from intended interventions	Incomplete outcome data	No bias in measurement of outcomes	Selective reporting	Other bias
Huh et al. (2021) [35]	?	+	?	?	-	?	?	?
Youssef and Paddle (2021) [55]	?	?	?	?	+	?	?	?
Syamal et al. (2021) [56]	+	-	-	?	?	-	?	?

Data items

Study characteristics included type of study, number of patients, intervention used, patient age, BMI, American Society of Anesthesiologists (ASA) grade, type of surgery, and neuromuscular blockade. Efficacy was defined by the following primary outcomes: duration of surgery, number of desaturations (defined as oxygen saturation (SpO2) <91% by the majority of the studies), and secondary outcomes including the need for rescue intervention (defined by conversion to intubation or jet ventilation), need for intubation, lowest SpO2, peak partial pressure of arterial carbon dioxide (PaCO2), peak venous partial pressure of CO2 (PvCO2), peak end-tidal carbon dioxide (EtCO2), peak transcutaneous CO_2_ (or values at end of case), rate of EtCO2 increase, pH, bicarbonate, and other complications. 

Data Collection Process

Two reviewers (KC and TY) screened the included studies independently, extracted the data and summarised it as a systematic review table. A third reviewer (KK) adjudicated if there were conflicts. Numerical data was extracted from diagrams, the appendices of the studies were checked, and authors were contacted for missing data. If a study did not explicitly discuss complications, we reported it as “not reported”. 

Synthesis Methods

We summarised effect estimates using descriptive statistics (median, interquartile range (IQR), and range) and applied a median effect estimate. For the comparative studies, the risk of bias table and meta-analysis were performed using the latest version of ReviewManager (RevMan) (5.3). Median and IQRs were converted to estimate mean and standard deviation using the method described by Wan et al. [57]. A random effects model (DerSimonian and Laird method) was utilised due to an anticipated high degree of heterogeneity. Pooled estimates for dichotomous outcomes were presented as risk ratios (RRs) with 95% confidence intervals (CIs) and for continuous outcomes, mean differences with standard deviations were used. Studies were assessed for heterogeneity using Cochran’s Q test and I2 tests. Substantial heterogeneity was defined as I2 > 75%, The risk of publication bias was assessed by funnel plots. Robustness tests were planned to investigate heterogeneity. Low heterogeneity and few studies precluded sensitivity analysis. TSA was performed to determine the reliability of the meta-analysis, utilising TSA software version 0.9 (The Copenhagen Trial Unit, Centre for Clinical Intervention Research, The Capital Region, Copenhagen University Hospital - Rigshospitalet, Copenhagen, Denmark) [58].

Certainty Assessment

Confidence in the results obtained was assessed by two authors independently (KC and TY) using the Grading of Recommendations, Assessment, Development and Evaluations (GRADE) framework [59]. The quality of evidence was rated very low, low, moderate, and high. Disagreements were adjudicated by the third author (KK). 

## Review

Results

*Characteristics of HFNC Studies* 

The characteristics of the 14 HFNC studies of 464 patients are summarised in Table 4 [40-53]. Ten studies were case series; 10 specified THRIVE, and others specified HFNC with or without humidified oxygen. The median age of the patients was 54.9 (IQR = 51-59.5, range = 26-63). ASA was reported in 12 studies; 44 patients were ASA 1, 160 patients were ASA 2, and 83 patients were ASA 3. The median BMI was 26.8 (IQR = 25.1-28.8, range = 24.8-31). Six studies were of microlaryngoscopy, five were of laryngotracheal procedures, two were of subglottic procedures, and one was of upper airway endoscopy. Muscle relaxant was used in 11 studies, not used in one study, and not reported in two. 

**Table 4:** Characteristics of HFNC studies ASA: American Society of Anesthesiologists; RCT: randomized controlled trial; HFNC: high-flow nasal cannula; THRIVE: Transnasal Humidified Rapid-Insufflation Ventilatory Exchange; STRIVE Hi: SponTaneous Respiration using IntraVEnous anaesthesia and Hi-flow nasal oxygen; SV: spontaneous ventilation; NR: not reported

Study (First author, date)	Type of study	Intervention studied	Number of patients (n)	Patient age	ASA grading	BMI	Type of surgery	Neuromuscular block used	Gender
Benninger (2021) [40]	Case series	THRIVE	53	51 (19-75) (median, range)	ASA 1 = 3 ASA 2 = 24 ASA 3 = 21 ASA 4 = 5	25 (19-50) (median, range)	Microlaryngeal surgery	Rocuronium	Female: 33/53 Male: 20/53
Booth (2017) [41]	Case series	STRIVE Hi	27	55 (46-65) (31-78)) yrs (SD, range)	ASA 3 =30	29 (19–46)	Laryngotracheal surgery	NR	NR
Booth (2020) [42]	RCT	HFNC (SV vs apneic)	19 (SV= 9 apnea = 10)	SV: 62.9 (9.1) Apnea: 58.1 (12.6) (mean, SD)	SV ASA 2 = 7 ASA 3 =2 Apnea ASA 2= 9 ASA 3 = 1	SV 28.8 (25.2–30.0) Apnea 26.6 (23.4–29.1) (median, IQR)	Microlaryngoscopy Panendoscopy	Rocuronium	NR
Ebeling (2019) [43]	Case series	THRIVE	3	Case 1:59 Case 2: 26 Case 3 60	NR	NR	Microlaryngoscopy Microdebridement of VC	Rocuronium	NR
Flach (2019) [44]	Case series	HFNC	21	NR	NR	NR	Cordectomy for subglottic stenosis	NR	Male: 9/21 Female: 12/21
Yang (2019) [45]	Case Series	THRIVE	23	54.8 (16) (mean, SD)	ASA 2 =22 ASA 3= 1	25.8 (27.3- 22.5) (median, IQR)	Microlaryngoscopy	Cisatracurium and suxamethonium	Male: 14 Female: 9
Huang (2019) [46]	Case series	THRIVE	56	No rescue ventilation: 60 (39-70) Rescue Ventilation: 57 ( 45- 66) (median, IQR)	ASA 2 = 44 ASA 3= 12	No rescue Ventilation: 26 (23 to 30) Rescue ventilation 31 (29 to 33) (median, IQR)	Microlaryngoscopy including laser	Rocuronium, vecuronium or mivacurium	NR
Lyons (2017) [47]	Case Series	HFNC	28	56.6 (18) (mean SD)	ASA 1=8 ASA 2= 15 ASA 3= 5	24.8 (4.5) (mean, SD)	Laryngeal / tracheal surgery	Rocuronium	Female: 14/28 Male: 14/28
Maupeu (2018) [48]	Case Series	THRIVE	19	49 ( 26-76)	2 (1-3)	25 (18-35) (median, range)	Laryngoscopy	None used	NR
Patel and Nouraei (2015) [49]	Case series	THRIVE	25	49 (15 (25–81) (mean, SD, range)	ASA grade was 3 (2–3 (1–4)) (median, IQR, range)	30 (23–36 (18–52)) (median, IQR, range)	Hypopharyngeal and laryngotracheal surgery	Rocuronium	NR
Rutt et al. (2022) [50]	Cohort	HFNC	38	63 (14.3, 36-89) (mean, SD, range)	ASA 1=13 ASA 2 = 14 ASA 3= 9 ASA4 1	27.2 (5, 17.6-37.5) (mean, SD, range)	Laryngotracheal procedures	Not specified	Female: 24/38 Male: 14/38
To (2017) [51]	Case series	THRIVE	17	52( 20-74)	ASA 2= 15 ASA 3 = 2	27 (20-36) (mean, range)	Balloon dilatation for subglottic stenosis	Not specified	Male: 2 Female: 8
Waters (2019) [52]	Case series	THRIVE	105	53.63 (17.49) ( mean, SD)	Median ASA 2 (1-4)	27.04 (5.04) (mean, SD)	Upper airway endoscopy	Not specified	NR
Gustafsson (2017) [53]	RCT	HFNC (Hyperventilation vs non-hyperventilation)	30	51 (12.7) 27-76	ASA 1= 20 ASA 2= 10	25.1 (3.5) (mean, SD)	Microlaryngoscopy Short laryngeal procedures	Rocuronium	Female: 9/30 Male: 21/30

Outcomes of HFNC Studies 

The outcomes of HFNC studies are summarised in Table 5. The duration of surgery was reported by two studies; 12.4 (4.4) minutes by Yang et al. [48] and 20.8 (7.8) minutes by Gustafsson et al. [56]. The duration of apnea was reported by 12 studies, the median was 21.7 minutes (IQR = 17-24.55, range = 10-30). The number of desaturations was reported by 12 studies and the median rate of desaturation was 8.1% (IQR= 4.55-16.6, range = 0-34.2). The total number of desaturations was 53 out of 448, and the need for rescue intervention was reported by 12 studies, with a median percentage for rescue intervention of 14.2% (IQR = 3.3-22.6, range= 2.9-100). The total number of rescue interventions was 60 out of 405. The need for intubation was reported by 11 studies, median percentage for intubation was 3.9% (IQR 1.9-19.6, range = 0-100). The total number of intubations was 38 out of 383 (mean = 9.9%). The lowest SpO2 or SpO2 after the end of apnea was reported by nine studies, median 91% (IQR = 76-95.25, range = 60-100). The peak PaCO2 or PaCO2 at the end of apnea was reported by three studies, median 10.2 kpa (range 9.44-11.86). The peak EtCO2 or EtCO2 at the end of apnea was reported by eight studies, median 7.4 (IQR = 7.025-7.9, range = 6.66-8.47). The peak transcutaenous CO2 was reported by two studies, ranging from (9.33-11.92). The rate of EtCO2 increase was reported by eight studies, the median was 0.15 kPa/min (IQR = 0.12-0.16, range = 0.03-0.17). pH at the end of apnea was reported by three studies, the median was 7.2 (range = 7.11-7.23). Bicarbonate at the end of apnea or the end of the case was reported by three studies, the median was 23.5 mmol/L(range 23.5-25.4). 

**Table 5:** Outcome of HFNC pH: potential of hydrogen; SpO2: oxygen saturation; CO2: carbon dioxide; EtCO2: End-tidal carbon dioxide; PaCO2: arterial partial pressure of carbon dioxide; PvCO2: venous partial pressure of carbon dioxide; ASA: American Society of Anesthesiologists; RCT: randomized controlled trial; HFNC: high-flow nasal cannula; THRIVE: transnasal humidified rapid-insufflation ventilatory exchange; STRIVE Hi: SponTaneous Respiration using IntraVEnous anaesthesia and Hi-flow nasal oxygen; SV: spontaneous ventilation; NR: not reported; IQR: interquartile range; SD: standard deviation

Study (First author, date)	Duration of surgery (min)	Apnea time (min)	Number of desaturation / total cases (%)	Need for rescue intervention / total cases (%)	Need for intubation / total number of cases (%)	Lowest SpO2 / SpO2 at end of apnea (%)	Peak PaCO2 / PaCO2 at end of apnea or peak PvCO2 (kPa)	Peak EtCO2 or values at end of case (kPa)	Peak transcutaenous CO2 (kPa)	Rate of EtCO2 increase (kPa/min)	pH at end of apnea or end of case	Bicarbonate at end of apnea or end of case (mmol/L)	Complications (intraoperative and postoperative)
Benninger (2021) [40]	NR	16 (4-42) (median, range)	12/53 (22.6)	12/53 (22.6)	6/53 (11.3)	95 (75-100)	NR	6.66 (3.0-10.1) (median, range)	NR	0.113	NR	NR	Bronchospasm = 1
Booth (2017) [41]	NR	NR	3/30 (10)	1/30 (3)	1/30 (3)	100 (99–100 (97–100))	NR	6.8 (6.4–7.1 (4.8-8.9)) kPa. (median, IQR)	NR	0.03	NR	NR	NR
Booth (2020) [42]	NR	30	NR	NR	NR	NR	Apnea: 11.86 SV: 7.36 kpa	NR	NR	NR	Apnea: 7.11 (0.04) SV 7.29 (0.06) (mean, SD)	Apnea = 23.5 SV = 25	NR
Ebeling (2019) [43]	NR	NR	NR	3/3 (100)	3/3 (100)	NR	NR	NR	Case 1: 9.33 Case 2: 9.33 Case 3: 11.92	NR	NR	NR	NR
Flach (2019) [44]	NR	15-95 min	1/21 (4.8)	NR	NR	NR	NR	NR	NR	NR	NR	NR	NR
Yang (2019) [45]	12.4 (4.4) min. (mean, SD)	24.1 (6.4) min. (mean, SD)	1/23 (4.3)	1/23 (4.3)	1/23 (4.3)	72	NR	NR	NR	NR	NR	NR	Nil
Huang (2019) [46]	NR	25 (20-28) (median, range)	12/56 (21.4)	12/56 (21.4)	11/56 (19.6)	60	NR	NR	NR	NR	NR	NR	NR
Lyons (2017) [47]	NR	20.9 (7.4) (mean, SD)	2/28 (7.1)	4/28 (14.2)	1/28 (3.5)	95.5 (4.5) (mean, SD)	9.44 (1.1) (mean, SD) +	8.47 (1.7) (mean, SD)	NR	0.17	7.23 (0.04) (mean, SD)	25.4 (1.8) (mean, SD)	NR
Maupeu (2018) [48]	NR	27 (11) (mean, SD)	2/22 (9.1)	4/22 (18.2)	NR	NR	NR	NR	NR	NR	NR	NR	Laryngospasm = 1 Hypercapnia = 1
Patel (2015) [49]	NR	14 (9–19 (5–65)) min.	0/25 (0)	19/25 (surgically required) (76)	4/25 (surgically required) (16)	NR	NR	7.8 (2.4 (4.9–15.3)) kPa.	NR	0.15	NR	NR	Spasmodic dysphonia = 1
Rutt (2022) [50]	NR	23.9(6.1, 13-40) (mean, SD, range)	13/38 (34.2)	17/38 (44.7)	13/38 (34.2)	93.6 (6.1, 79-100) (mean, SD, range)	NR	7.25 (1.58, 2.66-10.7) (mean, SD, range)	NR	0.15 (0.06, 0.05-0.34) (mean, SD, range)	NR	NR	Nil complications reported
To (2017) [51]	NR	18( 10-27) (median, range)	2 /17 (11.8)	2/17 (11.8)	0/17 (0)	80	NR	7.4 (5-8.7) (median, range)	NR	0.17	NR	NR	Nil complications reported
Waters (2019) [52]	NR	20 min ( 2-60) (median, range)	5/105 (4.8)	3/105 (2.9)	2/105 (1.9)	89.5 (70-91) (median, range)	NR	8 (4.4-16) (median, range)	NR	0.13	NR	NR	NR
Gustafsson (2017) [53]	20.8 (7.8) (mean, SD)	22.5 (4.5) (mean, SD)	0/30 (0)	1/30 (Supraglottic Jet) (3.3)	0/30 (0)	91	10.2 (1.4) (mean, SD)	7.4 (1.2) (mean, SD)	10.3 (1.6) (mean, SD)	0.15	7.2 (0.05) (mean, SD)	23.5 (22.5-24.5) (median, IQR)	NR

Complications were reported by six studies. There were four complications out of 178 patients (Mean = 2.2%); one case of bronchospasm, one case of laryngospasm, one case of hypercapnia, and one case of spasmodic dysphonia. No cases of airway fire were reported. 

Characteristics of Jet Ventilation Studies 

The characteristics of the 21 jet ventilation studies of 6336 patients are summarised in Table 6 [11-32]. There were 16 case series, three comparative studies, and two prospective cohorts. Nineteen studies reported the patient’s age, two studies did not report the patient’s age, and one study only reported the range. For the reported studies, the median age was 50.6 (IQR = 48-58 range = 41.5-67.5). The ASA was reported in 13 studies; 617 patients were ASA 1, 986 patients were ASA 2, 355 patients were ASA 3, and three patients were ASA 4. The median BMI reported in seven studies was 26.9 (IQR = 25.7-28.4, range = 24.8-34). Fourteen studies were of microlaryngeal surgeries, others were of laryngeal surgery and robotic laryngeal surgery. Muscle relaxant was used in 15 studies, not reported in five, and not used in one study. 

**Table 6:** Characteristics of jet ventilation studies NR: not reported; SHFJV: superimposed high-frequency jet ventilation; HFJV: high frequency jet ventilation; NFJV: normal frequency jet ventilation; TTJV: transtracheal jet ventilation; TGJV: transglottic jet ventilation; HFJV: High frequency jet ventilation; TIVA: total intravenous anaesthesia; NMB: neuromuscular blocker; MV: mechanical ventilation; AIV: apneic intermittent ventilation; CFJV: combined frequency jet ventilation

Study (First author, date)	Type of study	Intervention studied	Number of patients (n)	Patient age	ASA grading	BMI	Type of surgery	Neuromuscular block used	Gender
Leiter (2012) [23]	Case series, comparative study	SHFJV, NFJV, HFJV (supraglottic), HFJV (infraglottic)	16	49.8 (33-72)	ASA 1=7 ASA 2= 9	NR	Laryngeal surgery	Rocuronium	NR
Barakate (2010) [15]	Case Series	Non jet: 16 Supraglottic Jet 243 Infraglottic Jet: 741	1000	10-89 (range)	NR	NR	Microlaryngeal surgery	NR	NR
Bacher (2000) [14]	Case series, comparative study	Monofrequency Jet ventilation vs combined frequency Jet ventilation	23	HF 47.4 (18.6) LF 50.9 (17.9)	ASA 1-3	NR	Microlaryngeal surgery	Vecuronium	NR
Mora (2017) [24]	Case series	HFJV vs Intubation	62 HFJV= 14 Intubation =48	67.5 (median)	NR	NR	Microlaryngeal surgery	NR	Male : 56 Female : 6
Patel (2009) [26]	Case Series	Jet ventilation	352	NR	NR	NR	Suspension microlaryngoscopy	Not specified	NR
Rezaie-Majd (2006) [28]	Case series	SHFJV	1515	48.5(20) (mean, SD)	ASA 1-3	NR	Endoscopic larnygotracheal surgery	Vecuronium	NR
Halmos (2020) [18]	Cohort	SHFJV	163 patients, 224 procedures	Non-Converted group 58 (17) Converted group 61(15)	Non converted group ASA 1,2 =155 ASA 3,4= 43 Converted group ASA 1,2 =14 ASA 3,4= 11	Non converted group 28 (6) Converted group 34 (5)	Upper airway surgery	NR	Male: 103 Female: 95
Lanzenberger-Schragl (2000) [22]	Case series	SHFJV	500	48.5 (19.4)	NR	NR	Laryngeal, tracheal surgery	Vecuronium	NR
Bacher (2000) [13]	Case Series	Subglottic vs supraglottic+ subglottic and supraglottic CFJV	37	Group 1 = 50 (13) In Group 2 = 45 (17) (mean, SD)	ASA 1-3	NR	Microlaryngeal surgery	Vecuronium	NR
Rubin (2005) [30]	Case series	Subglottic jet ventilation	142	41.5 (16.7) (mean, SD)	NR	NR	Suspension Microlaryngoscopy	NR	NR
Bourgain (2001) [16]	Case series	High frequency Jet ventilation for endoscopic airway surgery	643	54 (13) (mean, SD)	NR	NR	laryngoscopy, laryngeal laser surgery	Suxamethonium infusion Vecuronium	Male: 523 Female: 120
Altun (2018) [12]	Case series	Infraglottic Jet catheter	222 With complication = 53 Without complications = 169	With complication 48.9 (11.7) Without complication 52.6 (11.2)	With complication ASA 1= 18 ASA 2 = 26 ASA 3 = 9 Without complication ASA 1= 60 ASA 2= 98 ASA 3= 11	With complication = 27.23 (4.14) Without complication = 25.49 (3.07) (mean, SD)	Microlaryngeal surgery	Mivacurium	Male: 149 Female: 73
Davies (2009) [17]	Cohort	Subglottic hunsaker mon jet tube	552	48 ( 17) (mean, SD)	ASA 1= 112 ASA 2= 343 ASA 3= 94 ASA 4= 3	BMI >30 = 92 BMI >40 = 7	Microlaryngeal surgery	Suxamethonium Vecuronium	Male: 348 Female: 204
Krespi (2019) [21]	Case series	Transnasal intubation = 22 Transoral intubation= 27 Jet ventilation = 8	52	47 (14)	NR	NR	Robotic surgery	NR	NR
Jaquet (2006) [20]	Cohort	TTJV vs TGJV vs AIV vs MV	1093 procedures in 661 patients, MV = 200 Intermittent apneic ventilation= 159 Transtracheal Jet ventilation = 265 Transglottic jet ventilation= 469	MV 31.5 (27.1) AIV 17.6 (23.8) TTJV = 52.1 (19) TGJV= 48.3 (21.6) (mean, SD)	ASA 1 = 358 ASA 2 = 575 ASA 3 = 160	NR	Microlaryngoscopy	Vecuronium Suxamethonium	NR
Putz (2022) [27]	RCT	Comparing TIVA with or without NMB	33 Deep NMB = 18 No NMB = 15	Deep NMB 61.1 (9.5) No NMB 60.3 (12.7) (mean, SD)	NR	Deep NMB 26 (5) No NMB 26 (6) (mean, SD)	Laryngeal transoral surgery	Rocuronium	Male : 22 Female: 11
Helmstaedter (2015) [19]	Case series	Single subglottic catheter	97	59 (19-88) (median, range)	ASA 1= 23% ASA 2 = 61% ASA 3 = 15% ASA 4 = 1%	26.6 (mean)	Microsurgies of larynx and trachea	Mivacurium	NR
Russell (2000) [31]	Case series	Cricothyroidotomy and transtracheal ventilation	90	55 (16) (mean, SD)	ASA 1 = 26 ASA 2 = 36 ASA 3 = 12 ASA 4 = 0	NR	Microsurgies of larynx	Not specified	Male: 36 Female: 47 Not recorded: 5
Abad (2007) [11]	Case series	Percutaneous transtracheal JV	42	43.54 (12.04) (mean, SD)	ASA 1-2	24.8 (2.78) (mean, SD)	Microlaryngoscopy	Atracurium	Male: 28 Female: 14
Ross-Anderson (2011) [29]	Case series	Transtracheal jet ventilation	50	60 (21-88) (median, range)	ASA 3 or above	NR	Microlaryngoscopy surgery in severe airway compromise and stridor	Atracurium	NR
Orloff (2002) [25]	Cohort	Subglottic Jet ventilation vs standard ETT	84	NR	ASA 1 and 2 = 73 ASA 3= 11	NR	Microlaryngoscopy	NR	NR

Outcomes of Jet Ventilation Studies 

The outcomes of jet ventilation studies are summarised in Table 7. Duration of surgery was reported by six studies, median time 53 minutes ( IQR= 41-74, range =20-95). Duration of apnea was reported by three studies, with one study providing a range of 20-110 minutes and two studies giving a range of 27.2-45 minutes. The number of desaturations was reported by seven studies of 3328 patients. There were 71 events in total, with a median rate of desaturation of 2.25% (IQR= 0.9-3.9, range 11.4). The need for rescue intervention was reported by seven studies with 1997 patients, with 74 events in total, and a median percentage for rescue intervention of 2.7% (IQR = 2.3-10.3, range= 0.3-11.6). The need for intubation was reported by 10 studies of 4186 patients, with 70 events in total, and a median percentage for intubation of 1.2% ( IQR 0.3-6.9, range = 0.2-11.6 ). The lowest SpO2 or SpO2 after the end of apnea was reported by two studies, ranging from 50% to 95%. The peak PaCO2 or PaCO2 at the end of apnea was reported by six studies, median 5.65 kpa (IQR= 5-5.9, range 4-7.3). The peak EtCO2 or EtCO2 at the end of apnea was reported by two studies, ranging from 4.3 kpa to 7.5 kpa. pH at the end of apnea was reported by one study with a result of 7.345+/- 0.037. The peak transcutaneous CO2, rate of EtCO2 increase and bicarbonate at the end of apnea or at the end of the case were not reported by any studies. 

**Table 7:** Outcomes of jet ventilation studies SpO2: oxygen saturation; PaCO2: arterial partial pressure of carbon dioxide; PvCO2: venous partial pressure of carbon dioxide; EtCO2: end-tidal carbon dioxide; CO2: carbon dioxide; pH: potential of hydrogen; CPAP: continuous positive airway pressure; HDU: high dependency unit; MV: manual ventilation; AIV: apneic intermittent ventilation; JV: jet ventilation; NR: not reported; SD: standard deviation; Bx: biopsy

Study (First author, date)	Duration of surgery (min)	Apnea time (min)	Number of desaturation / total cases (%)	Need for rescue intervention / total cases (%)	Need for intubation / total cases (%)	Lowest SpO2 / SpO2 at end of apnea	Peak PaCO2 / PaCO2 at end of apnea or peak PvCO2	Peak EtCO2 or values at end of case	Peak transcutaenous CO2	Rate of EtCO2 increase	pH at end of apnea or end of case	Bicarbonate at end of apnea or end of case	Complications (intraoperative and postoperative)
Leiter (2012) [23]	NR	NR	NR	NR	NR	NR	NR	NR	NR	NR	NR	NR	Nil complications reported
Barakate (2010) [15]	NR	NR	NR	NR	NR	NR	NR	NR	NR	NR	NR	NR	Required CPAP in HDU overnight = 3
Bacher (2000) [14]	NR	45	NR	NR	23/23	NR	NR	NR	NR	NR	NR	NR	NR
Mora (2017) [24]	NR	NR	NR	NR	NR	NR	NR	NR	NR	NR	NR	NR	NR
Patel (2009) [26]	NR	20 - 110	3/352 (0.9)	1/352 (0.3)	1/352 (0.3)	NR	NR	NR	NR	NR	NR	NR	Inadvertent oesophageal placement = 1
Rezaie Majd (2006) [28]	NR	NR	3/1512 (0.2)	NR	3/1512 ( 0.2)	NR	5.6 (1.3) (mean, SD)	NR	NR	NR	NR	NR	Nil complications reported
Halmos (2020) [18]	NR	NR	26/224 (11.6)	26/224 (11.6)	26/224 (11.6)	NR	NR	NR	NR	NR	NR	NR	Nil complications reported
Lanzenberger (2000) [22]	NR	27.2 (16.1) (mean, SD)	12/500 (2.4)	12/500 (2.4)	3/500 (0.6)	NR	4 (0.7) (mean, SD)	NR	NR	NR	NR	NR	Nil complications reported
Bacher (2000) [13]	NR	NR	NR	NR	NR	NR	PaCo2 > 7.3 seen in 38.8% of subglottic LFJV 16.2% in subglottic CFJV 5.3% in supraglottic CFJV	NR	NR	NR	NR	NR	NR
Rubin (2005) [30]	NR	NR	NR	NR	NR	NR	5 (0.89) (mean, SD)	NR	NR	NR	NR	NR	Esophageal placement = 1
Bourgain (2001) [16]	NR	NR	25/643 (3.9)	NR	2/643 (0.3)	NR	NR	NR	NR	NR	NR	NR	Subcutaneous emphysema = 67 Pneumomediastinum = 16 Pneumothorax = 7
Altun (2018) [12]	Direct laryngoscopic examination and Bx 20 (15-40) Microlaryngeal resection 20 (20-45) Cordectomy- dilatation 45(15-60)	NR	NR	5/222 (2.3)	10/222 (4.5)	NR	NR	NR	NR	NR	NR	NR	Bradycardia = 6 Hypotension = 24 Arrhythmia = 4
Davies (2009) [17]	55 (31) (mean, SD)	NR	NR	15/552 (2.7)	15/552 (2.7)	NR	NR	NR	NR	NR	NR	NR	Stridor = 1
Krespi (2019) [21]	NR	NR	NR	NR	NR	NR	NR	NR	NR	NR	NR	NR	Nil complications reported
Jaquet (2006) [20]	NR	NR	NR	NR	NR	NR	NR	NR	NR	NR	NR	NR	MV = nil complications, Minor complications = 29 (AIV), Major complications = 4 Larygnospasm = 5 (AIV) Barotrauma = 7 (JV) Failed insertion or failed ventilation cannula = 7 Mucosal damage = 1 Laryngospasm = 1 Hemodynamic instability= 8 Myocardial infarction = 1 Subcutaneous emphysema= 4 Pneumothorax = 3
Putz (2022) [27]	Deep NMB 74 (50) No NMB 95 (88) (mean, SD)	NA	NA	NA	NA	NA	NA	NA	NA	NR	NR	NR	Nil complications reported
Halmstaedter (2015) [19]	53 (3 - 303) (mean, range)	NR	2/97 (2.1)	10/97 (10.3)	9/97 (9.3)	50%	NR	NR	NR	nR	NR	NR	Nil complications reported
Russell (2000) [31]	NR	NR	NR	NR	NR	NR	NR	7.5 ( 1.4) (mean, SD)	NR	NR	NR	NR	12/90 Cricothyroid puncture related = 3 Surgical emphysema = 2 Bleeding = 1 Bradycardia = 1 Regurgitation= 2 Kinking = 1
Abad (2007) [11]	NR	NR	NR	NR	NR	NR	5.7 (0.5) BMI <25 5.2 (0.3) BMI >25 6.2 (0.2) (mean, SD)	NR	NR	NR	7.345(0.037) BMI <25 7.37 (0.01) BMI >25 7.311 (0.023) (mean, SD)	NR	Nil complications reported
Ross-Anderson (2011) [29]	NR	NR	0	10% ( n=5)	NR	NR	NR	NR	NR	NR	NR	NR	10 complications Minor bleeding, Surgical emphysema Catheter kinking = 4
Orloff L A (2002) [25]	41 (15- 100) (mean, range)	NR	NR	NR	1/84 (1.2)	95	5.9	4.3	NR	NR	NR	NR	Laryngospasm = 1

There were 184 complications reported by 10 studies of 4587 patients (mean = 4.01%). There were 73 cases of surgical emphysema, 32 cases of hypotension and hemodynamic instability, 16 cases of pneumomediastinum, 10 cases of pneumothoracies, eight cases of bradycardia, and four cases of arrhythmia. There were three cases which required CPAP post operatively and stayed in a high dependency unit overnight, 2 cases of esophageal placement, 7 cases of failure to insert cannula, 3 cases of cricothyroid puncture, 1 case of mucosal damage, 1 case of laryngospasm, 1 case of stridor, 1 case of bleeding, 1 case of kinking of the catheter. No cases of airway fire were reported. 

Characteristics of Comparative Studies 

The characteristics of the comparative studies are summarised in Table 8 [32-35,54-56]. For the comparative studies, we labeled the patients undergoing HFNC as the THRIVE group (all were paralysed). There were seven comparative studies of 521 patients with 206 patients in the THRIVE group (39.5%), four of which were randomised controlled studies, three were retrospective reviews. There were four studies of THRIVE versus intubation and mechanical ventilation, one of THRIVE versus jet ventilation and intubation, one of THRIVE versus standard pre-oxygenation, and one of THRIVE versus low-flow oxygenation. The median age of patients undergoing THRIVE was 54.45 years (IQR = 48.2-59, range = 47-59), comparable to the median age of patients undergoing other forms of ventilation (55.5 years; IQR = 51.3-58, range = 43-58). The ASA grading was reported in six studies. In the THRIVE group, 20 patients were ASA 1, 90 patients were ASA 2, 65 patients were ASA 3, and nine patients were ASA 4. In the standard ventilation group, 36 patients were ASA 1, 81 patients were ASA 2, 74 patients were ASA 3, and 19 patients were ASA 4. The BMI of patients undergoing THRIVE was 25.9 (IQR = 23.8-27.1, range = 23.2-29.6), and the BMI of patients undergoing standard ventilation was 26.4 (IQR = 24.7-31.2, range = 24.7-33.0). All studies were of patients undergoing laryngeal microsurgery. All studies used muscle relaxation. 

**Table 8:** Characteristics of comparative studies NR: not reported; IQR: interquartile range; SD: standard deviation; RCT: randomized controlled trial; ETT: endotracheal tube; MV: mechanical ventilation; AAIV: apnea with intermittent ventilation; LFO: low flow oxygen

Study (First author, date)	Type of study	Intervention studied	Number of patients (n)	Patient age	ASA grading	BMI	Type of surgery	Neuromuscular block used
Forsberg (2020) [32]	RCT	THRIVE vs mechanical ventilation (MV)	THRIVE = 15 MV = 15	THRIVE = 48.2 (19.9) MV = 51.3 (12.3) (mean, SD)	THRIVE ASA 1= 7 ASA 2= 8 MV ASA 1 = 11 ASA 2 = 4	THRIVE = 25.99 (4.5) MV = 25.95 (3.9) (mean, SD)	Microlaryngoscopy	Rocuronium
Min (2022) [33]	Randomized non-inferiority study	HFNC VS tracheal intubation	HFNC = 56 Tracheal intubation = 62	HFNC= 59 (20-84) Tracheal intubation =58 ( 25-78) (median, IQR)	HFNC ASA 1= 12 ASA 2 =39 ASA 3 = 5 Tracheal intubation ASA 1= 23 ASA 2 =38 ASA 3 = 1	HFNC = 23.8 (3.5) Tracheal intubation = 24.7 (3.0)	Laryngeal microsurgery	Rocuronium
Nekhendzy (2020) [34]	Prospective, randomized, patient-blinded, 2-arm parallel pilot trial	TI/SHFJV VS THRIVE	n=20 THRIVE= 10 TI/SHFJV=10	THRIVE= 50.9 (12.4) TI/SHFJV 55.2 (17.3) (mean, SD)	THRIVE ASA 1 =1 ASA 2 = 8 ASA 3 = 2 TI/SHFJV ASA 1 =2 ASA 2 = 5 ASA 3= 3	THRIVE = 25.8 (4.8) TI/SHFJV 26.4 (5.1) (mean, SD)	Laryngeal microsurgery MDL excision / Bx VC injection Balloon dilatation	Rocuronium
Huh (2021) [35]	Retrospective chart review	THRIVE vs ETT	140 10: concurrent operations 23: have laser 9: converted ventilation THRIVE:44 ETT: 54	58 (mean, range)	NR	ETT: 24.7 (2.68) THRIVE: 23.2 (3.03) (mean, SD)	Microlaryngoscopy	Rocuronium
Rajan (2018) [54]	Randomized single-blinded study	THRIVE vs Standard preoxygenation and nasopharyngeal oxygenation	n= 10 THRIVE = 5 Standard = 5	THRIVE = 38.0 (27 - 78.5) Standard = 48 (37.0 - 63.0) (median, IQR)	THRIVE ASA 1 = 3 ASA 2 = 2 Standard ASA 1 = 1 ASA 2 = 4	NR	Direct laryngoscopy	Atracurium
Youssef (2021) [55]	Comparative review	THRIVE vs Low flow oxygenation	n= 35 THRIVE = 12 LFO= 23	THRIVE 47 (17) LFO 43 (13.5) (median, IQR)	THRIVE ASA 2= 8 ASA 3= 4 LFO ASA 2= 9 ASA 3= 14	THRIVE 27.1 (8.6) LFO 31.2 (8.5) (mean, SD)	Microlaryngoscopy	Nondepolarizing neuromuscular blocker
Syamal (2021) [56]	Retrospective cohort study	Intubated vs intermittently intubated (AAIV) vs fully apneic with Jet, THRIVE or HFNC (10-15L/min)	n=200 Intubated: (n= 52) Apnea with intermittent ventilation ( AAIV): (n=58) Jet 4/58 HFNC 29/58 Fully apneic:(n=90) Jet 2/90 HFNC 51/90 THRIVE 8/90	Intubated 55.5 (42.5–67.0) AAIV 55.5 (43.0–68.0) Fully apneic 59.0 (48.0–71.0) (median, IQR)	Intubated ASA 1= 0 ASA 2= 17 ASA 3=26 ASA4 = 9 AAIV ASA 1= 0 ASA 2= 8 ASA 3= 40 ASA4 = 10 Fully apneic ASA 1=0 ASA 2=27 ASA 3=54 ASA4 =9	Intubated 29.0 (25.5–33.5) AAIV 33.0 (29.0–37.2) Fully apneic 29.6 (26.3–33.9) (median, IQR)	Microlaryngeal surgery	Succinylcholine Rocuronium

*Outcomes of Comparative Studies* 

The outcomes of the comparative studies were summarised in Table 9 [32-35,54-56]. The duration of surgery was reported by five studies. Duration of apnea was reported by all studies, number of desaturations was reported by six studies, need for rescue intervention was reported by five studies, and need for intubation was reported by five studies. The lowest SpO2 or SpO2 at the end of apnea was reported by all studies. Peak ETCO2 was reported by three studies. Peak PaCO2, peak transcutaneous CO2, rate of EtCO2 or PaCO2 increase, pH at the end of apnea, and complications were reported by two studies. Bicarbonate at the end of the case was reported by one study. Only five of the comparative studies [33-35,57,58] could be included in the meta-analysis. We performed meta-analysis on the duration of surgery, number of desaturations, need for rescue intervention, lowest SpO2, and peak EtCO2. In the five studies included in the meta-analysis, standard ventilation referred to tracheal intubation and SHFJV, with only four patients in Nekhendzy et al.'s study undergoing SHFJV [34]. 

**Table 9:** Outcomes of comparative studies NR: not reported; IQR: interquartile range; SD: standard deviation; RCT: randomized controlled trial; ETT: endotracheal tube; HFNC: High-flow nasal cannula; THRIVE: transnasal humidified rapid-insufflation ventilatory exchange; SHFJ: super high frequency jet ventilation; MV: mechanical ventilation; AAIV: apnea with intermittent ventilation; LFO: low flow oxygen; TI: tracheal intubation

Study (First author, date)	Duration of surgery	Apnea time	Number of desaturation / total cases (%)	Need for rescue intervention / total cases (%)	Need for intubation / total number of cases (%)	Lowest SpO2 / SpO2 at end of apnea	Peak PaCO2 / PaCO2 at end of apnea or peak PvCO2 (mmHg or kPA)	Peak EtCO2 or values at end of apnea	Peak transcutaenous CO2	Rate of EtCO2 increase / rate of PaCO2 increase (kPa/min)	pH at end of apnea or end of case	Bicarbonate at end of apnea or end of case (mmol/L)	Complications (intraoperative and postoperative)
Forsberg (2020) [32]	THRIVE = NR MV = 28.1 (12.8) (mean, SD)	THRIVE = 17.9 (4.8) MV = NA (mean, SD)	THRIVE = 3/15 (20) MV = 0/15 (0)	THRIVE = 3/15 (20) MV = 0/15 (0)	THRIVE = 3/15 (20) MV = 0/15 (0)	THRIVE = 96.1 (4.7) MV = 97.2 (1.3) (mean, SD)	PaCO2 THRIVE = 9.8 (1.3) MV = 5.3 (0.7) (kpa) (mean, SD)	THRIVE = 7.6 (1.1) MV = 4.7 (0.6) (kPA, mean, SD)	NR	PaCO2 THRIVE = 0.28 (0.10) EtCO2 THRIVE = 0.17 (0.08) MV = 0 (mean, SD)	THRIVE = 7.2 (0.05) MV = 7.4 (0.04) (mean, SD)	NR	NR
Min (2022) [33]	HFNC= 15 (10-25) Tracheal intubation 20 (15-30) (median, IQR)	HFNC 20 (15-30) 21.76 (11.41) (mean, SD) Tracheal intubation NR	HFNC 3/56 (5.4) Tracheal intubation 0/62 (0)	HFNC 13/56 (23.2) Tracheal 2/62 (3.2)	NR	HFNC= 100 (98-100) Tracheal 100 (100-100) (median, IQR)	NR	HFNC 6.8 (5.6- 8.8) Tracheal 4.9 (4.8- 5.2) (median, IQR)	HFNC 7.8 (6.7-8.6) Tracheal 6.1 (5.7-6.4) (median, IQR)	HFNC 0.12	NR	NR	NR
Nekhendzy (2020) [34]	THRIVE 19.1 (10.8) TI/SHFJ 20.9 (8.8) (mean, SD)	THRIVE 29.9 (10.4) TI/SHFJ 39.2 (12.7) (mean, SD)	THRIVE 0/10 (0) TI/SHFJ 0/10 (0)	THRIVE 0/10 TI/SHFJ 0/10	THRIVE 0/ 10 TI/SHFJ 6/10	THRIVE = 93 (5.6) TI/SHFJ = 98.7 (1.6) (mean, SD)	NR	NR	NR	NR	NR	NR	NR
Huh (2021) [35]	THRIVE = 16.3 (9.69) min ETT = 21.9 (12.0) min,	NR	THRIVE: 1/44 (0.7) ETT 0/54	NR	NR	THRIVE 97.9 (2.51) ETT: 98.0 (1.76)	NR	NR	11.0 THRIVE: 10.9 ETT: 7.33	NR	NR	NR	NR
Rajan (2018) [54]	NR	THRIVE 796 (43.36), 780 (760 - 840) Standard 444 (52.56) 420 (300 - 600) (seconds) (mean, SD) (median, IQR)	THRIVE 0/5 (0) Standard 4/5 (80)	THRIVE 0/5 (0) Standard 4/5 (80)	THRIVE 0/5 (0) Standard 0/5 (0)	THRIVE 99.4 (1.34) 100 (98.5 - 100) Standard 85.80 (9.3) 86.0 (77 - 94.5) (mean, SD) (median, IQR)	THRIVE 69.46 (7.15) 66.4 (65.8 - 74.65) Standard 59.00 (4.64) 59.4 (54.45 - 63.15) (mean, SD) (median, IQR)	NR	NR	NR	THRIVE 7.21 (0.03) 7.21 (7.18 - 7.24) Standard 7.23 (0.04) 7.24 (7.18 - 7.25) (mean, SD) (median, IQR)	THRIVE 24.4 (2.99) 24.60 (21.4 - 27.3) Standard 23.08 (3.82) 23.4 (19.25 - 26.58) (mean, SD) (median, IQR)	NR
Youssef (2021) [55]	NR	THRIVE 35.3 (24) LFO 31.6 (14.8) (mean, SD)	THRIVE 0/12 LFO 14/23 (60.9)	THRIVE 4 /12 (33.3) LFO 14 (60.9)	0/35 (0)	THRIVE: 90 LFO: 75	NR	NR	NR	NR	NR	NR	Nil complications reported
Syamal (2021) [56]	Intubated 28.5 (20.0–41.0) AAIV 20.5 (13.0–42.0) Fully Apneic 10.0 (5.0–33.0) (median, IQR)	Intubated 0 AAIV 5.0 (3.0–9.0) Fully Apneic 4.0 (2.0–8.0) (median, IQR)	NR	NR	NR	Intubated 91.0 (79.5–94.0) AAIV 81.0 (73.0–84.0) Fully apneic 92.0 (86.0–97.0) (median, IQR)	NR	Intubated 6.4 (5.7–7.5) AIIV 6.5 (5.9–7.5) Fully apenic 4.4 (4.0 –5.1) (median, IQR)	NR	NR	NR	NR	Intubated Dysphonia = 2 Hemorrhage = 1 CVS event = 1 Tooth/ oral injury = 2 AIIV dysphonia = 1 Dysphagia = 1 Dyspnea = 6 Hemorrhage = 1 Death = 1 Pulmonary event = 1 Pain = 4 Fully apneic Dysphonia = 5 Dyspnea = 4 Hemorrhage = 2 Laryngeal edemia = 1 Cardiovascular event = 1 Tooth/ oral injury = 2 Pain = 4

**Figure 2:**
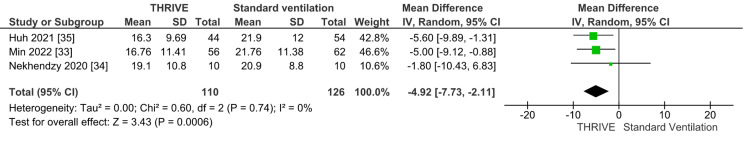
Duration of surgery forest plot SD: standard deviation; CI: confidence interval; IV: inverse variance; THRIVE: transnasal humidified rapid-insufflation ventilatory exchange

Three studies [33-35] including 236 patients reported the duration of surgery as an outcome. The duration of surgery was significantly decreased in the THRIVE group as show in Figure 2 (OR -4.92, 95%CI -7.73 to -2.11) (GRADE: Moderate). There was insignificant heterogeneity with an I^2 of 0%, Z-value (p = 0.0006). 

**Figure 3:**
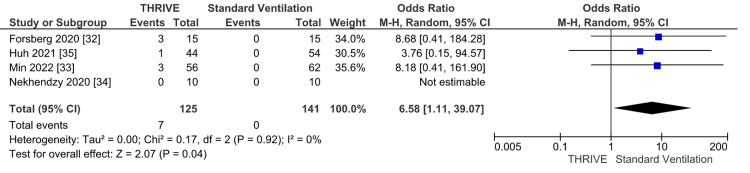
Number of desaturation forest plot M-H: Mantel-Haenszel; CI: confidence interval; THRIVE: transnasal humidified rapid-insufflation ventilatory exchange

Four studies [32-35] including 266 patients reported the number of desaturations intraoperatively as an outcome. Although a rare event, the number of desaturations was significantly lower in the standard ventilation group as shown in Figure 3 (OR 6.58 95%CI 1.11 to 39.07). There was insignificant heterogeneity with an I^2 of 0%, Z-value (p = 0.04). 

**Figure 4:**
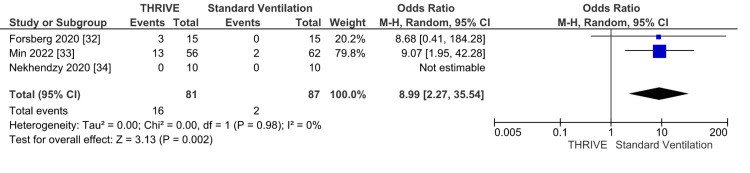
Need for rescue intervention forest plot M-H: Mantel-Haenszel; CI: confidence interval; THRIVE: transnasal humidified rapid-insufflation ventilatory exchange

Three studies [32-34] including 168 patients reported the need for rescue interventions intraoperatively as an outcome. The need for rescue intervention was significantly lower in the standard ventilation group as reported in Figure 4 (OR 8.99, 95%CI 2.27 to 35.54) (GRADE: Moderate). There was insignificant heterogeneity with an I^2 of 0%, Z-value (p = 0.002). 

**Figure 5:**
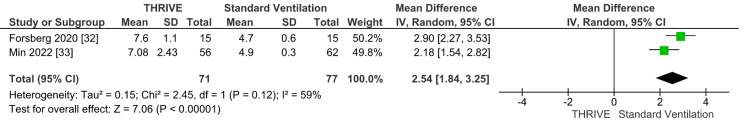
Peak EtCO2 forest plot SD: standard deviation; CI: confidence Interval; IV: inverse variance; EtCO2: end-tidal carbon dioxide; THRIVE: transnasal humidified rapid-insufflation ventilatory exchange

Two studies [32,33] including 148 patients reported the peak EtCO2 as an outcome. The peak EtCO2 was significantly higher in the THRIVE group as shown in Figure 5 (OR 2.54, 95%CI 1.84 to 3.25) (GRADE: Moderate). There was moderate heterogeneity with an I^2 of 59%, Z-value (p < 0.00001).

Visual examination of the funnel plots found no evidence of publication bias for all comparisons (see Appendix). 

**Figure 6:**
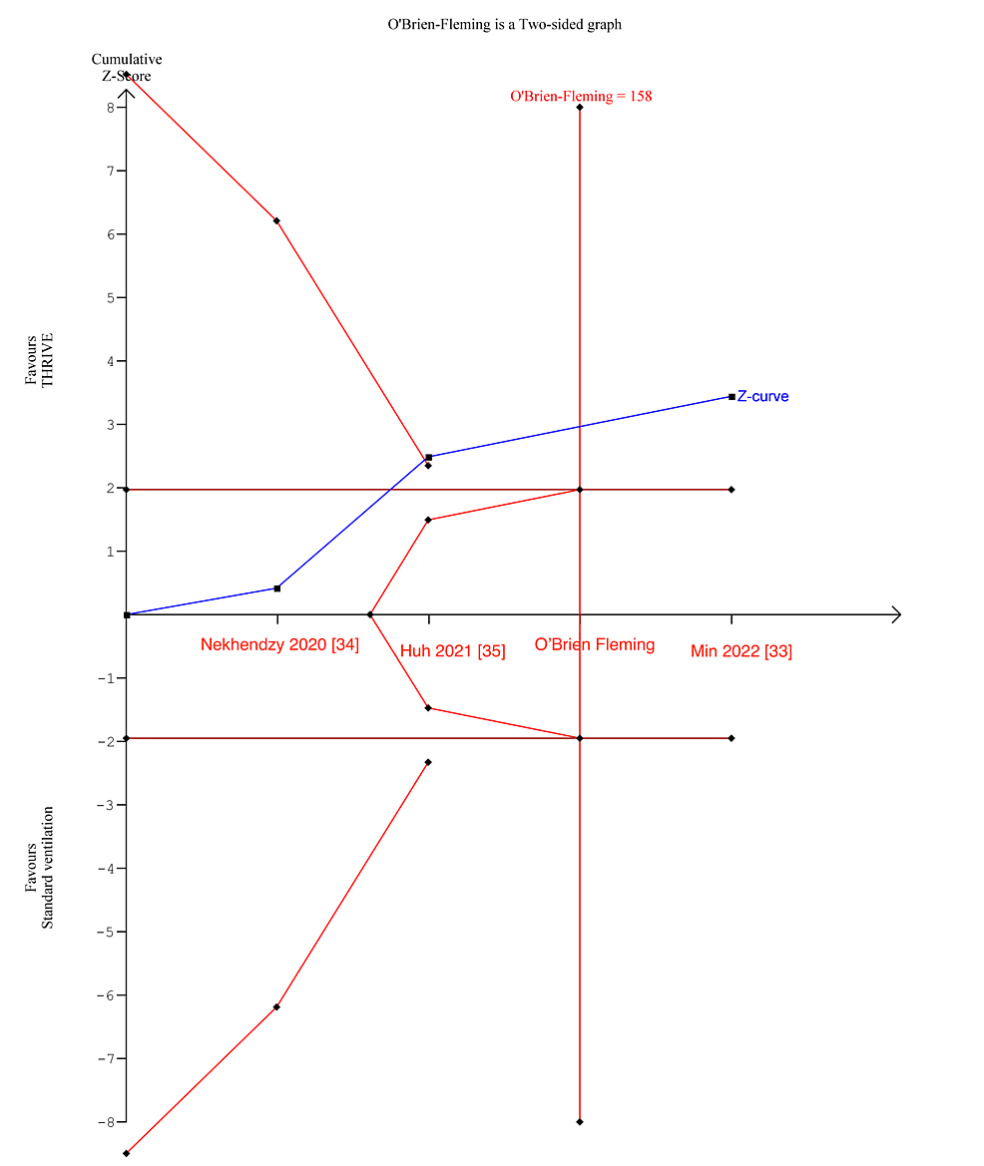
Duration of surgery TSA TSA: trial sequential analysis

TSA is a methodology which weighs type I and II errors and regards the addition of each trial in the meta-analysis as an interim meta-analysis [41]. This helps to quantify the statistical reliability of data and assess the need for further trials [60]. In Figure 6, for the duration of surgery, the Z-curve (blue line) crossed the boundary for conventional benefit, the monitoring boundary, and exceeded the estimated information size, indicating there was sufficient statistical power.

**Figure 7:**
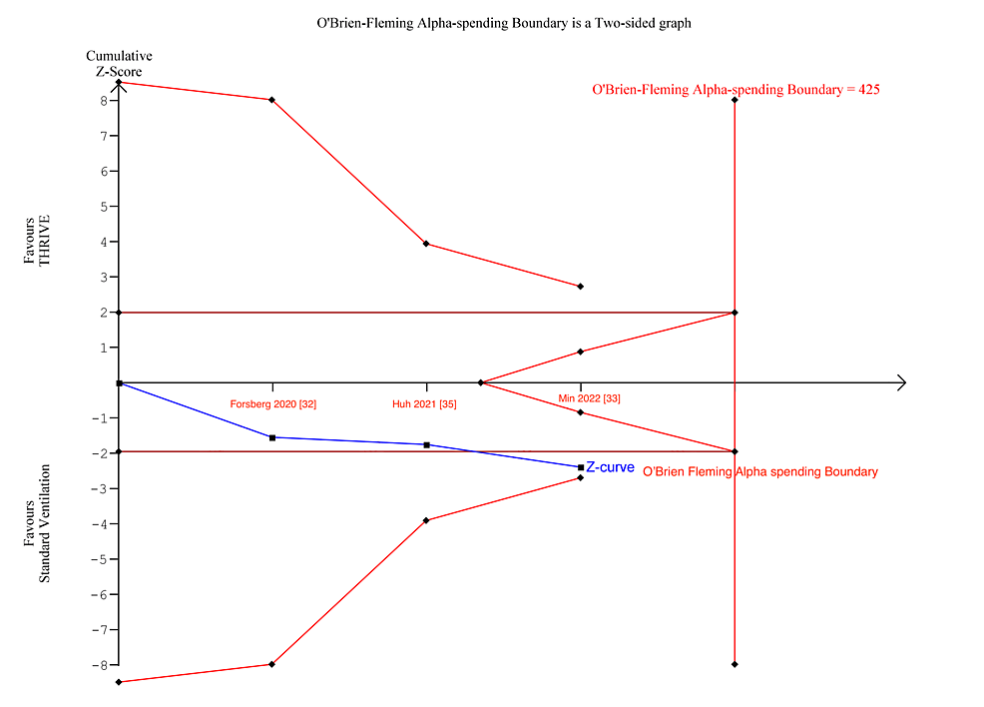
Number of desaturations TSA TSA: trial sequential analysis

**Figure 8:**
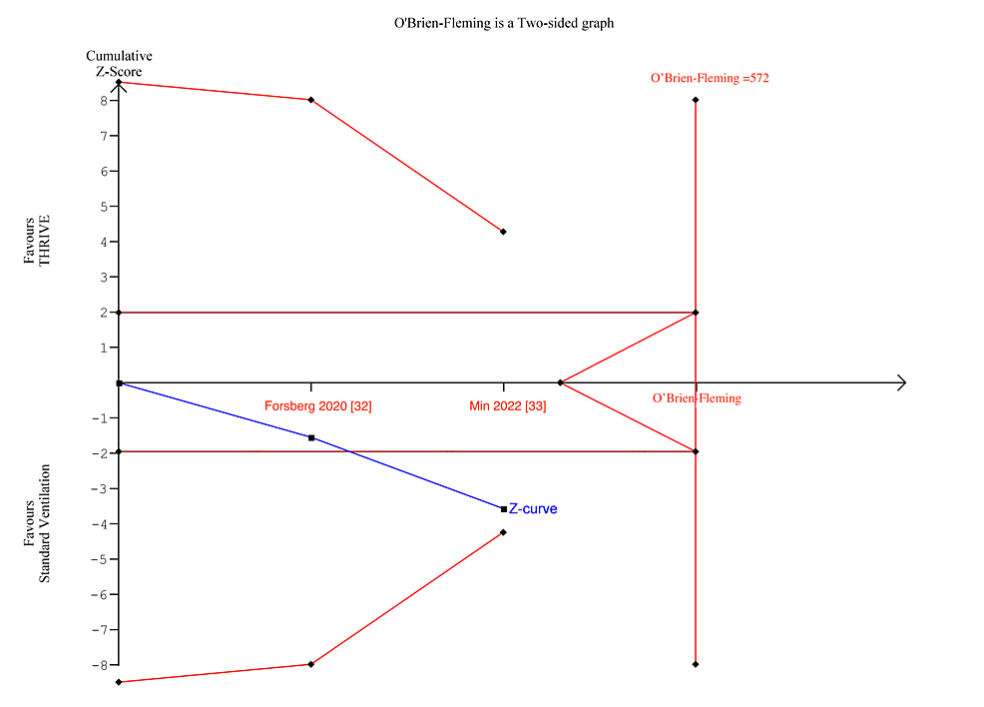
Number of rescue interventions TSA TSA: trial sequential analysis

In Figures 7-8, for number of desaturations and rescue interventions, the z-curve crosses the boundary for conventional benefit and monitoring boundary, but does not exceed the information size, indicating the meta-analysis findings were significant but lacked insufficient statistical power.

**Figure 9:**
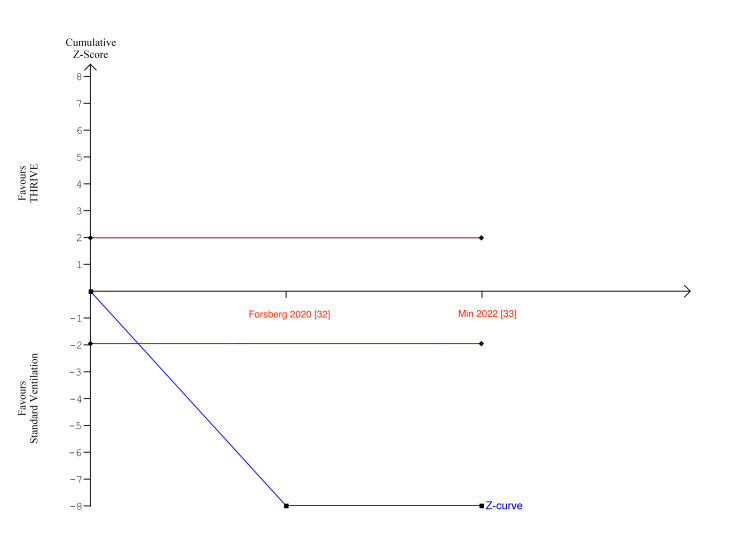
Peak EtCO2 TSA TSA: trial sequential analysis; EtCO2: end-tidal carbon dioxide

In Figure 9, the O’Brien-fleming alpha-spending boundaries were not renderable as the first information fraction already exceeded 100% of the required information size, indicating that conventional ventilation significantly reduced the peak EtCO2 with sufficient statistical power. 

Discussion

The use of HFNC in the intraoperative setting as an alternative to mechanical and jet ventilation has been gaining in popularity, especially for shared-airway procedures such as endoscopy, bronchoscopy, and laryngeal procedures [61-64]. While there are prior meta-analyses and systematic reviews of the use of HFNC in the induction period or for gastroscopy and bronchoscopy [61,62,64], this pre-registered study is the first that focuses on laryngeal surgery, comprehensively compares HFNC and conventional ventilation, systematically reviews jet ventilation in the setting of laryngeal surgery, and includes a trial sequential analysis. In the meta-analysis of comparative studies comparing THRIVE to standard ventilation, the duration of surgery was reduced in the THRIVE group (Figure 2), and the information size was reached in the TSA indicating sufficient statistical power. There were significantly fewer desaturation events (Figure 3), need for rescue intervention (Figure 4), and a lower peak EtCO2 (Figure 5) in the standard ventilation group. The studies included in the meta-analyses were found to have low risk of selection bias. Complications and mortality were rare in both groups. 

The patient populations were similar between both the HFNC and jet ventilation sets of case series, with a median age of 54.9 (HFNC) and 50.6 (jet ventilation), with a majority of ASA 1 and 2, and median BMI of 26.8 and 26.9, respectively. The profile of interventions was similar, with the majority of studies looking at microlaryngeal and laryngeal surgery. In most studies, the median apnea time was between 15-20 minutes. After this period, transcutaenous CO_2_ and PaCO2 increased but desaturation was rare. The median rate of desaturation was 8.1% in the HFNC studies and 2.25% in the jet ventilation studies, and the need for rescue intervention was 14.2% in the HFNC studies and 2.3% in the jet ventilation studies. The median peak PaCO2 was 10.2 kpa in the HFNC studies and 5.65 kpa in the jet ventilation studies, indicating better gas exchange. The mean rate of complications in the HFNC studies was 2.2% and 4.0% in the jet ventilation studies. Complications of jet ventilation were more serious, including surgical emphysema, pneumomediastinum and pneumothorax. The clinical consequences of a higher EtCO2 or decreased intraoperative SpO2 were not documented. 

Analyses by Liu et al. [62] and Spence et al. [63] showed that the minimum O_2_ saturation was higher and safe apnea time was extended in the HFNC group compared to oxygenation by regular nasal cannula. Hung et al.’s study of patients undergoing gastrointestinal endoscopy showed that HFNC was associated with reduced risks of oxygen desaturation, severe hypoxemia, other airway interventions, procedure interruption, and CO_2_ level but did not affect procedure time [61]. Our study shows for short laryngeal procedures, HFNC is a viable strategy for apneic oxygenation and reduces the duration of surgery, hypothetically due to an improved surgical view and turnover time by reducing the need for intubation and extubation. 

The studies included in this review and meta-analysis were mostly of elective surgeries performed on ASA 1 and 2 patients with a median age of 54.9 and a median BMI of 26.8. Although Lee and Quek reported a case of the use of THRIVE in a morbidly obese patient to facilitate airway surgery [65], obese patients have reduced functional residual capacity, higher risk of lung atelectasis, obstructive sleep apnea, respiratory depression, reflux and regurgitation. While the use of THRIVE is safer than pre-oxygenation or low-flow oxygen [61-63], our study shows there is an increased risk of desaturation, number of rescue interventions, and peak EtCO2 compared to mechanical ventilation and SHFJV. In patients with risks of aspiration and laryngospasm, less physiological reserve, pulmonary hypertension, and severe obstructive sleep apnea, and the morbidly obese, MV with a definitive airway remains the potentially safer option. 

Laser is occasionally used in laryngeal surgery and airway fire is a potentially fatal complication. Several of the studies in both the HFNC and jet ventilation groups included patients undergoing laser surgery [15,46,35], with no cases of airway fire reported. Some studies have reported the use of THRIVE during laser cases without adverse events or airway fire by turning off the oxygen flow for 40 seconds before the use of laser or by decreasing the fraction of inspired oxygen and suctioning airway gases [5,46]. In a physical model simulation, laser use in high-flow nasal oxygen can lead to “violent” self-sustained fires with continuous laser [66]. Further precautions such as using low-wattage lasers, minimizing lasering time, and reducing oxygen concentration should be taken to minimize the risks of combustion when utilizing lasers with THRIVE [67,68], but the short use of diathermy was safe, although there has been a case report of intra-oral ignition of monopolar diathermy during the use of THRIVE [68].

Limitations 

Most of the data in this study was largely dependent on the results of non-comparative case series and retrospective studies. There were only a few comparative studies and fewer RCTs. In the retrospective comparative studies, the grouping of the interventions and patients made meta-analysis impossible decreasing the amount of analysable data [54-56]. Publication bias was unavoidable as negative results are less likely to be submitted or accepted for publication. The jet ventilation studies were older compared to HFNC studies, introducing bias. We reduced this risk by only including recent studies, utilizing the Joanna Briggs Institute critical appraisal checklist for case series and only including well-performed case series with reliable data. Many of the older jet ventilation studies did not report the outcomes, reducing the amount of aggregatable data. There were several studies that did not report or discuss complications; however, this was true for both HFNC and jet ventilation studies, which likely reduced any effect on the safety analysis. Summarising the effect estimates by using the median and IQR, while an accepted method according to the Cochrane Handbook, is limited as it does not account for differences in the sizes of the studies and its performance has not been evaluated. 

The statistical heterogeneity in the meta-analysis was low, but the number of studies included was low and had a moderate to high risk of bias; however, the risk of selection bias was low for the comparative studies included in the meta-analyses as seen by the risk of bias assessments. We increased the reliability of our results through trial sequential analyses, implementing strict inclusion/exclusion criteria reducing the clinical heterogeneity and utilizing the GRADE framework. While the standard ventilation arm included SHFJV patients, the number was low (n=4) so there was minimal risk of introducing a confounder. For the retrospective comparative studies, there was a risk of patient selection bias in choosing conventional ventilation or HFNC. This impact was minimized as the meta-analysis consisted mostly of prospective RCTs and only one retrospective study, which had a low risk of selection bias [35]. Despite the differences, the impact on length of hospital stay, mortality, or complication rate compared to conventional ventilation is uncertain. 

## Conclusions

This pre-registered, comprehensive study shows that while HFNC is a viable and safe alternative in adult laryngeal surgery including microlaryngoscopy, suspension laryngoscopy, and laryngotracheal procedures, conventional ventilation may be safer. Compared to conventional ventilation, HFNC was associated with reduced operative time, but with an increased risk of desaturation, hypercarbia, and requirement for rescue intervention. Leaving aside the benefits of tracheal intubation, apnoeic HFNC oxygenation should be used with cautious monitoring during laryngeal microsurgery. The use of HFNC for laryngeal surgery settings mandates close communication between anesthesiologists and surgeons for patient selection and rescue interventions and patient-specific rescue plans should be instituted. In higher-risk patients, conventional ventilation with endotracheal intubation remains the gold standard from a safety perspective.

Larger prospective RCTs of HFNC versus jet ventilation and HFNC versus MV in laryngeal surgery are needed, with a focus on the postoperative outcomes and consequences of desaturation and high EtCO2. Further research on the selection of certain groups of patients who are safe for HFNC such as those with high BMI, elderly, pediatrics, and patients with obstructive sleep apnea is required. 

## Figures and Tables

**Figure 10:**
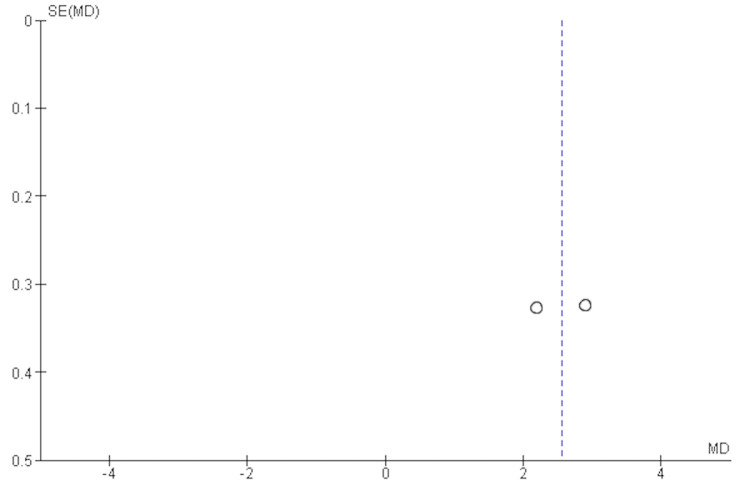
Peak EtCO2 funnel plot SE: standard error; MD: Mean difference; EtCO2: end-tidal carbon dioxide

**Figure 11:**
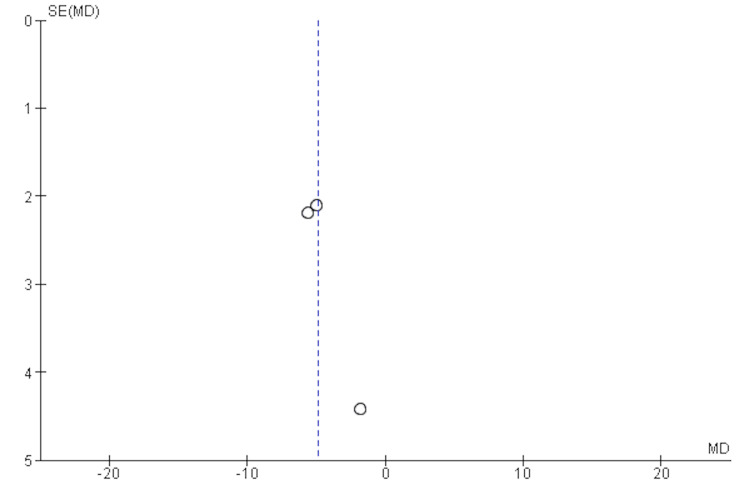
Duration of surgery funnel plot SE: standard error; MD: mean difference

**Figure 12:**
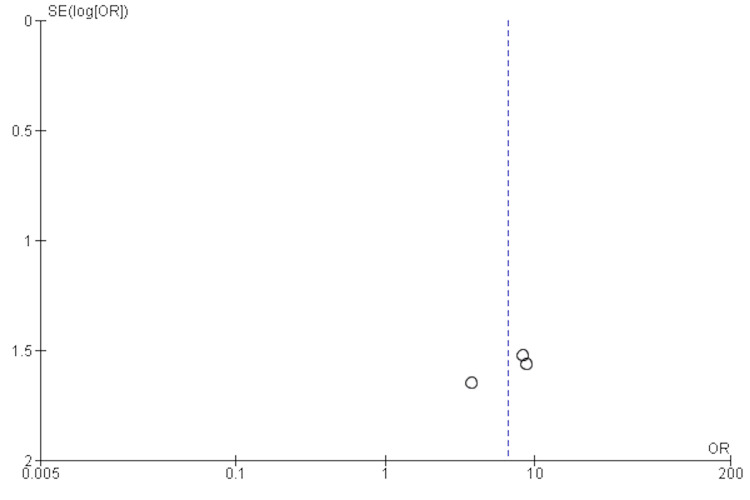
Number of desaturation funnel plot SE: standard error; OR: odds ratio

**Figure 13:**
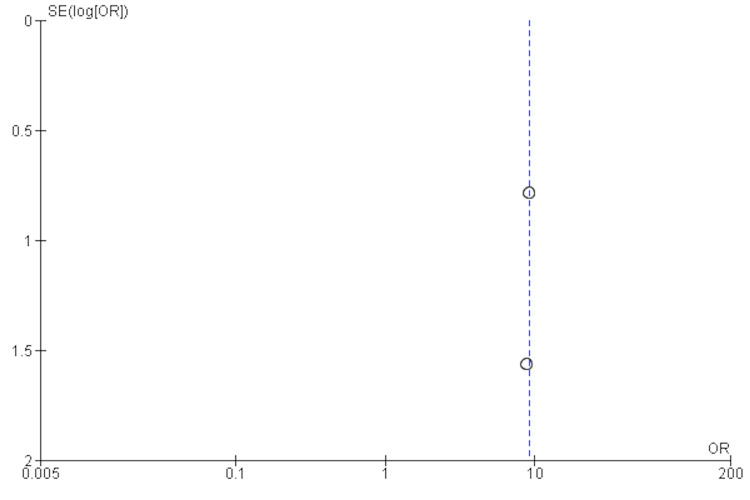
Need for rescue intervention funnel plot SE: standard error; OR: odds ratio

**Figure 14:**
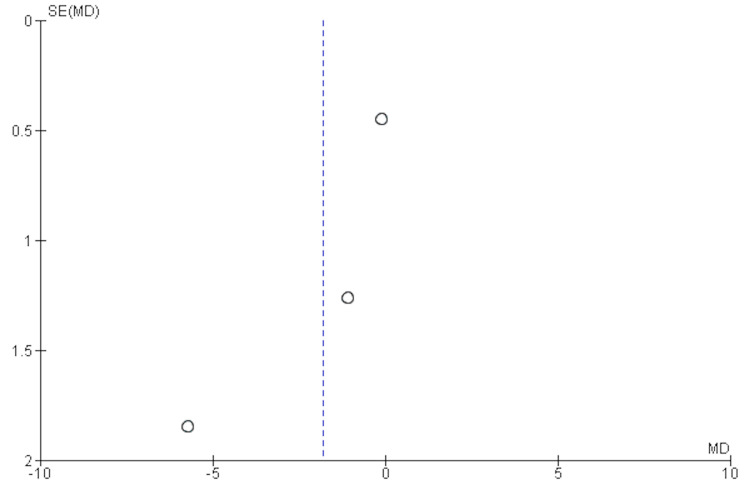
Lowest SpO2 funnel plot SE: standard error; MD: mean difference; SpO2: oxygen saturation
